# Exploring associations between the Phthalate Environmental Reproductive Health Literacy (PERHL) scale & biomarkers of phthalate exposure: A pilot study

**DOI:** 10.1038/s41370-024-00706-6

**Published:** 2024-07-17

**Authors:** Kathryn S. Tomsho, Marlee R. Quinn, Emma V. Preston, Gary Adamkiewicz, Tamarra James-Todd

**Affiliations:** 1 Department of Environmental Health, Harvard T.H. Chan School of Public Health, Boston, MA USA; 2 Department of Epidemiology, Harvard T.H. Chan School of Public Health, Boston, MA USA

**Keywords:** environmental health literacy, phthalates, biomarkers of exposure, psychometric scale

## Abstract

**Background:**

Perinatal exposure to phthalates is associated with adverse health impacts for parents and children. The field of environmental health literacy seeks to measure how environmental health information is conceptualized and used to inform behaviors. We assessed whether scores on the validated Phthalate Environmental Reproductive Health Literacy (PERHL) scale were associated with biomarkers of phthalate exposure.

**Methods:**

42 members of the Environmental Reproductive and Glucose Outcomes (ERGO) cohort completed the PERHL scale and provided spot urine samples. Phthalate summary measures for model outcomes were created by calculating molar sums of specific gravity-corrected metabolite concentrations representing exposure to parent phthalate, Di(2-ethylhexyl)phthalate (DEHP), personal care product (PCP)-associated phthalates, and parent butyl-phthalates. Linear regression models were used to estimate the associations of the PERHL scale scores with phthalate summary measures, controlling for educational attainment (college degree or higher vs. no college degree), age (years), and race and ethnicity (non-Hispanic White vs. non-White).

**Results:**

Higher scores on the PERHL Scale and subscales were generally associated with lower ΣDEHP, Σbutyl, and ΣPCP metabolite concentrations. A one-point increase in the ‘Protective Behavior/Risk Control’ subscale score was significantly associated with a −30.3% (95% CI: −50.1, −2.6) decrease in ΣDEHP, and a −30.6% (95% CI: −51.5, −0.63) decrease in Σbutyl metabolite concentrations.

## Introduction

Phthalates are a group of endocrine disrupting chemicals (EDCs) commonly used as plasticizers, in personal care products (PCPs), and in building materials [1]. Due to their ubiquity, over 95% of the United States population has detectable urinary concentrations of phthalate metabolites [2]. Perinatal exposure to phthalates is associated with a variety of adverse health outcomes for child and parent [1, 3, 4]. Further, phthalates can cross the placenta, resulting in fetal exposure that is strongly correlated with parental phthalate exposure [5]. For the child, perinatal exposures have been associated with preterm birth, low birth weight, short gestational length, and adverse effects in childhood such as neurodevelopmental effects [1, 6]. For the parent, exposure to phthalates during pregnancy has been associated with an increased risk of gestational diabetes, as well as hypertensive disorders of pregnancy [7–9]. Still, studies have found that exposure to phthalates can be reduced via behavioral and product use change, which can have implications for health across the life course for the parent and child [10–12]. Thus, developing tools to reduce perinatal phthalate exposures has advantages for parents and children across the life course.

The emerging field of environmental health literacy (EHL) seeks to evaluate and characterize how environmental health information is conceptualized, attended to, contextualized, and ultimately, used to inform behaviors [13–15]. Environmental health literacy is posited to be context and topic-specific, requiring the development of environmental health literacy measurement tools that are population-specific [13, 15]. To meet this goal, we previously developed and validated the Phthalate Environmental Reproductive Health Literacy (PERHL) Scale, which measures overall phthalate and reproductive health environmental health literacy [16]. Measurement via the PERHL Scale can assist in identifying individuals who may benefit from educational and behavioral environmental health literacy interventions [16]. Pregnancy is also a period of rapid behavior change in which there may be increased motivation to engage in behaviors that reduce phthalate exposures and their associated health effects.

To date, studies have not explored associations between environmental health literacy measurement tools and biomarkers of exposure, though there have been explorations of associations between environmental health literacy and exposures [17, 18]. However, developing tools that demonstrate correlation with biomarkers of relevant environmental exposures will contribute to the developing environmental health literacy toolkit by providing non-invasive means of identifying individuals who may be at risk of higher environmental exposures.

The objective of this pilot study was to explore associations between scores from the PERHL Scale and subscales with urinary phthalate metabolite concentrations among participants of the Environmental Reproductive and Glucose Outcomes (ERGO) cohort.

## Methods

The ERGO Study is a longitudinal pregnancy cohort focused on assessing the role of environmental exposures on reproductive and cardiometabolic health outcomes across pregnancy and the postpartum period. The details of ERGO study activities have been described elsewhere [19]. Briefly, ERGO recruited pregnant participants (*n* = 653 unique pregnancies contributed by *n* = 647 participants) without pre-existing diabetes from December 2016 to November 2020 from Boston, MA-area hospitals’ obstetrics clinics during routine prenatal visits [19]. Participant eligibility criteria included being at least 18 years of age, being less than 15 weeks of gestation at enrollment, and planning to receive prenatal care and deliver at a participating hospital. The ERGO cohort also includes participants enrolled in the Study of Pregnancy Regulation of INsulin and Glucose (SPRING) who contributed harmonized data during pregnancy and early postpartum but did not participate in the present study [20]. For this pilot study, all ERGO participants who were at least one year postpartum (*n* = 424) were invited to complete the PERHL survey. One hundred seventeen ERGO participants consented to complete the PERHL scale. Participants, all of whom had delivered within the last 5 years, completed the PERHL Scale remotely via REDCap between March 2021 and June 2022. Prior to completing the PERHL survey, ERGO participants did not engage in conversations about phthalates as part of their participation in the ERGO study. All participants were asked if they would like to opt-in to provide optional remote spot urine samples. Of the 117 participants who completed the PERHL Scale, 42 participants concurrently provided an optional remote spot urine sample. Participants collected remote urine samples using polypropylene sample containers shipped to them. Participants froze their samples until the time of shipment. All samples were shipped in insulated containers with ice packs via overnight shipping. All urine samples were received in appropriate conditions for analysis [21].

The PERHL Scale is a validated tool to assess environmental health literacy related to phthalate exposures during pregnancy. Overall scores range from 6 to 30, representing low to high environmental health literacy, respectively. In addition to the overall PERHL Scale score, there are 6 subscale scores capturing latent factors related to environmental health literacy: (1) ‘Awareness of Phthalate Reproductive Health Impacts’ (8 questions that address the extent to which respondents believe that exposure to phthalates may be associated with reproductive health impacts), (2) ‘Scientific Uncertainty’ (4 questions that address the extent to which respondents feel uncertain about the adverse effects of phthalates on environmental and human health due to various possible sources of uncertainty), (3) ‘Protective Behavior/Risk Control’ (5 questions addressing whether respondents try to minimize their use of certain personal care and household products due to concern of phthalate exposures, and to what degree they believe reducing their phthalate exposures is beneficial to their health), (4) ‘Regulatory Interest’ (3 questions addressing respondents’ belief that phthalates should be regulated based on their potential impacts to the environment and human health), (5) ‘Awareness of Phthalate Exposure Routes’ (3 questions addressing respondents’ understanding of sources of phthalates into the environment, and possible exposure pathways that may affect reproductive health outcomes), and (6) ‘General Phthalate Knowledge’ (3 questions very broadly addressing respondents’ belief that phthalates are in personal care products, and that people can be exposed to them by using personal care products). PERHL questions and the subscales they belong to are provided in Table 1.

**Table 1:** Summary of PERHL Subscales, Items, and Time to Completion.

Subscale	Estimated Time to Complete Section	Items
Awareness of Phthalate Reproductive Health Impacts	5 min	Reproductive health problems are possibly caused by things in the environment.
		Reproductive health problems are possibly caused by using personal care products (such as perfumes, deodorants, and soaps) that contain phthalates.
		Reproductive health problems for women trying to get pregnant are possibly caused by using personal care products (such as perfumes, deodorants, and soaps) that contain phthalates.
		Reproductive health problems for children are possibly caused by their mother’s exposure during pregnancy to personal care products (such as perfumes, deodorants, and soaps) that contain phthalates.
		One of the reasons for reproductive health disorders in humans (such as early puberty or birth defects in genitalia) is exposure to phthalates common in personal care products (such as perfumes, deodorants, and soaps) during pregnancy.
		I believe that there are chemicals in some personal care products (such as perfumes, deodorants, and soaps) that can affect human’s reproductive health.
		Phthalates may negatively impact the reproductive health of human beings.
		I believe that phthalates in common personal care products (such as perfumes, deodorants, and soaps) may cause reproductive health problems for humans.
Scientific Uncertainty	3 min	I am uncertain about the adverse effects of phthalates on humans and the environment because information is only available in certain sources.
		I am uncertain about the adverse effects of phthalates on humans and the environment because of insufficient attention and warning from scientists.
		I am uncertain about the adverse effects of phthalates on humans and the environment because of ineffective communication or difficulty understanding scientists’ communication about phthalates.
		I am uncertain about the adverse effects of phthalates on humans and the environment because reproductive health problems can be caused by things other than phthalates.
Protective Behavior/Risk Control	3 min	I think reducing my use of personal care products that may contain phthalates (such as certain perfumes, deodorants, and soaps) is important for my child’s health.
		I think reducing my use of personal care products that may contain phthalates (such as certain perfumes, deodorants, or soaps) is important for my health.
		I try to minimize my use of personal care products that may contain phthalates (such as certain perfumes, deodorants, or soaps).
		I try to minimize my use of plastic products because they may contain phthalates.
		I have concern about phthalates and their effects on humans although I try to avoid them in my personal care products.
Regulatory Interest	2 min	I think that phthalates should be regulated for their potential impact on the environment, in general.
		I think that phthalates should be regulated for their potential impact to human health.
		I believe that phthalates should be regulated for their potential impact on human health, even though their potential impacts are uncertain.
Awareness of Phthalate Exposure Pathways	2 min	Phthalates can enter into the environment from sources like personal care products (such as perfumes, deodorants, and soaps).
		Industrial processes that use phthalates may release them into the environment.
		Phthalates in personal care products (such as perfumes, deodorants, and soaps) may cause reproductive health problems for animals.
General Phthalate Knowledge	2 min	People can be exposed to phthalates by using personal care products (such as perfumes, deodorants, and soaps).
		Phthalates may have adverse health effects on human beings.
		I believe that phthalates are in some personal care products (such as perfumes, deodorants, and soaps).

Urine samples were analyzed for concentrations of 14 phthalate and 2 replacement phthalate metabolites, described in Table 2 [22] Samples were analyzed using previously described methods [23]. Urinary concentrations were specific-gravity (SG)-corrected to account for urinary dilution [24]. We created three phthalate summary measures for our model outcomes by calculating molar sums of SG-corrected metabolite concentrations representing exposure to: (1) parent phthalate, DEHP:$\sum DEHP=(\frac{mEHP}{278})+(\frac{mEHHP}{294})+(\frac{mEOHP}{292})+(\frac{mECPP}{308})$, (2) personal care product (PCP)-associated phthalates: $\sum PCP=(\frac{mBP}{222})+(\frac{mEP}{194})+(\frac{miBP}{222})$, and (3) parent butyl-phthalates: $\sum butyl=(\frac{mBP}{222})+(\frac{mBzP}{190})+(\frac{miBP}{222})$. Summary measure concentrations were log_2_-transformed due to skewed distributions and modeled as continuous outcomes.

**Table 2:** Summary of Measure Urinary Phthalate Metabolites and Abbreviations.

Phthalate	Abbreviation
Cyclohexane-1,2-dicarboxylic acid mono carboxyisooctyl ester	CX-MINCH
mono-2-ethyl-5- carboxypentyl terephthalate	MECPTP
mono-2-ethyl-5-hydroxyhexyl terephthalate	MEHHTP
Cyclohexane-1,2-dicarboxylic acid mono hydroxyisononyl ester	OH-MINCH
mono-n-butylphthalate	mBP
mono-benzyl phthalate	mBzP
mono-carboxy isononyl phthalate	mCNP
mono-carboxy octyl phthalate	mCOP
mono (3-carboxypropyl) phthalate	mCPP
mono-(2-ethyl-5-carboxypentyl) phthalate	mECPP
mono (2-ethyl-5-hydroxyhexyl) phthalate	mEHHP
mono ethyl hexyl phthalate	mEHP
mono (2-ethyl-5-oxohexyl) phthalate	mEOHP
monoethyl phthalate	mEP
mono-isononyl phthalate	mNP

We used linear regression models to estimate the associations of continuous PERHL Scale and subscale scores with concentrations of each phthalate summary measure, controlling for educational attainment (college degree or higher vs. no college degree), age (years), and race and ethnicity (non-Hispanic White vs. non-White) of the participant. The percent change in molar sums of phthalate metabolite concentrations was calculated from model estimates using the following formula: $\% {change}=({2}^{\beta }-1)* 100$. We additionally explored whether there were associations between education with the three summary phthalate measures.

Due to the small sample size in this pilot study, an alpha value of 0.1 was used as the significance threshold to account for the exploratory nature of the analysis and to minimize the risk of type II errors. All analyses were performed in R Software version 4.2.1.

## Results

Participant demographics are displayed in Table 3 for the present study population. Participants were mainly non-Hispanic White (71.8%), had a mean age of 33.1 years, and had a college degree or beyond (86.3%). This differed from the sociodemographic characteristics of participants in the full ERGO cohort, which had a lower percentage of non-Hispanic White participants (58%), and a lower percentage obtaining a college degree or beyond (79%). The mean age of the ERGO cohort was 32.9 years. Participants who opted in to provide urine samples (*n* = 42) were mainly non-Hispanic White (73.8%), had a mean age of 34.0 years, and had a college degree or beyond (88.1%).

**Table 3:** Participant Characteristics.

	ERGO (*n* = 653)	Pilot (*n* = 117)	Urine Sample (*n* = 42)
**Race/Ethnicity**^**a**^			
Non-Hispanic White	377 (57.9%)	84 (71.8%)	31 (73.8%)
Non-White	274 (42.1%)	22 (28.2%)	11 (26.2%)
**Age at Consent**			
Mean (SD)	32.9 ± 4.8	33.1 ± 4.3	34.0 ± 4.40
Median (Min, Max)	33.0 (19.3, 48.3)	32.0 (22.8, 42.9)	34.2 (22.8, 42.9)
**Education**^**b**^			
Less than College Degree	136 (21.5%)	16 (13.7%)	5 (11.9%)
College Degree or Beyond	497 (78.5%)	101 (86.3%)	37 (88.1%)

^a^*n* = 2 participants missing race and ethnicity data.

^b^*n* = 20 participants missing educational attainment data.

Table 4 displays the estimated percent difference in urinary concentrations of phthalate summary measures per 1-point increase in PERHL Scale & subscale scores. Higher scores on the PEHRL Scale and subscales were generally associated with lower ΣDEHP, Σbutyl, and ΣPCP metabolite concentrations. Higher PERHL Scale scores were significantly associated with lower ΣDEHP metabolite concentrations. Specifically, a one-point increase on the overall PERHL Scale was associated with a −7.2% (95% CI: −15.0, 1.3) decrease in ΣDEHP metabolite concentrations. Higher ‘Protective Behavior/Risk Control’ and ‘Regulatory Interest’ subscale scores were significantly associated with lower ΣDEHP and Σbutyl phthalate metabolite concentrations after adjustment for potential confounders. A one-point increase in the ‘Protective Behavior/Risk Control’ subscale score was significantly associated with a −30.3% (95% CI: −50.1, −2.6) decrease in ΣDEHP, and a −30.6% (95% CI: −51.5, −0.63) decrease in Σbutyl metabolite concentrations. Similarly, a one-point increase in the ‘Regulatory Interest’ subscale score was significantly associated with a −33.9% (95% CI: −53.5, −6.0) decrease in ΣDEHP, and a −28.4% (95% CI: −51.45, 5.4) decrease in Σbutyl metabolite concentrations. We found no evidence of an association between educational attainment and PERHL score or summary phthalate metabolite measures.

**Table 4:** Percent Difference in Urinary Phthalate Metabolite Concentrations per 1-point Increase in PERHL Scale & Subscale Scores.

	ΣDEHP	ΣPCP	Σbutyl
	concentrations	concentrations	concentrations
**Overall PERHL**	−7.2 (−15.0, 1.3) *	−2.5 (−14.1, 10.68)	−6.4 (−14.8, 2.9)
**Awareness of Phthalate Reproductive Health Impacts**	−6.9 (−35.3, 34.0)	−17.7 (−50.3, 36.2)	−9.0 (38.2, 34.1)
**Scientific Uncertainty**	−6.0 (−33.7, 33.3)	−8.8 (−44.0, 42.3)	−15.2 (−41.4, 22.6)
**Protective Behavior/Risk Control**	−30.3(−50.1, −2.6) **	−27.8 (−55.4, 17.0)	−30.6 (−51.5, −0.63) **
**Regulatory Interest**	−33.9 (−53.5, −6.0) **	−8.2(−45.7, 55.4)	−28.4 (−51.4, 5.4) *
**Awareness of Phthalate Exposure Routes**	−22.3 (−50.0, 20.8)	15.6 (−38.0, 115.7)	−6.4 (−42.0, 50.9)
**General Phthalate Knowledge**	−12.3 (−39.1, 26.4)	24.7 (−24.9, 106.9)	−3.8 (−35.0, 42.3)

*p < 0.1, **p < 0.05.

^a^controlled for age, race/ethnicity, and educational attainment.

^b^Values in the table represent the estimate and 95% confidence interval associated with a one-unit increase in the subscales (e.g., scientific uncertainty).

## Discussion

We found that higher scores on the validated PERHL Scale and subscales were overall associated with decreased phthalate metabolite concentrations. Scant literature has explored the relationship between environmental health literacy and biomarkers of exposure. One randomized controlled trial (*n* = 63) found that an environmental health literacy-based intervention was successful in improving indoor air quality, significantly reducing indoor PM2.5 (*z* = 56.39, *p* < 0.1) and volatile organic compounds (*z* = 10.11, *p* < 0.01) in the treatment group and resulted in significant differences between the treatment and control groups in the level of cotinine (*z* = −1.07, *p* = 0.15) and trans,trans-muconic acid (tt-MA) (z = −1.44, *p* = 0.31), bioindicators of exposure to the volatile exposures of interest [25]. Environmental health literacy was assessed via an adapted version of Lichtveld et al.’s environmental health literacy scale, which consists of a series of questions on air, water, and food as well as general environmental health literacy. All questions are related to environmental health knowledge, attitudes, or behaviors via Likert 5-point scale questions and summed, with higher scores indicating higher environmental health literacy. However, although environmental health literacy was assessed as a part of this study, the direct associations between environmental health literacy score and biomarkers of exposure were not investigated. The results of this study suggest that educational interventions can be effective at reducing indoor air pollutants and, further, reducing biomarkers of exposure.

Overall, the associations seen between higher PERHL scores and lower biomarkers of phthalate exposure support the hypothesis that environmental health literacy may be a protective factor against harmful environmental exposures that are amenable to behavioral interventions. Previous conceptualizations of environmental health literacy have hypothesized that increasing awareness or knowledge of a given environmental exposure leads to protective behavior, and subsequently, reduced exposures. However, the associations between higher scores on the ‘Awareness of Phthalate Exposure Routes’ and ‘General Phthalate Knowledge’ subscales suggest that assessment of environmental health literacy via measurement of knowledge on a topic, alone, may not be sufficient to capture the full scope of environmental health literacy dimensions. Future efforts to develop environmental health literacy measurement tools should explore latent factors beyond topical knowledge that contribute to environmental health literacy.

Additionally, we found higher ‘Protective Behavior/Risk Control’ scores were associated with significant decreases in ΣDEHP and in Σbutyl metabolite concentrations, but not ΣPCP metabolite concentrations. We hypothesize that this may be due to broad awareness and avoidance of phthalates in certain plastic, food packaging, and household products, but an unawareness of phthalates in personal care products. Additional research should explore the driver of the difference in associations across phthalate exposures sources.

Although the theory of environmental health literacy continues to evolve, it has been suggested that environmental health literacy of an individual is likely to be topic and context-specific. Levels of environmental health literacy will vary based on personal experiences and background relevant to a particular environmental exposure in various contexts [26]. Building tools to assess individual environmental health literacy related to specific contexts and environmental exposures is critical to ensure that environmental health literacy can be measured to identify opportunities for intervention to build environmental health literacy and facilitate actions aimed at the reduction of exposures, where possible. This work seeks to fill the gap by assessing the association between a validated environmental health literacy scale and biomarkers of phthalate exposure. Previous work has found that increasing environmental health literacy related to endocrine-disrupting chemicals can facilitate exposure-reduction behaviors [27]. Thus, developing non-invasive tools to assess environmental health literacy that are associated with physical indicators of exposure to the chemical of concern can provide a non-invasive means of identifying individuals who may benefit from educational interventions [27].

This study has several limitations. This pilot study has a small sample size, with only 42 participants opting to provide remote urine samples. This limits the generalizability of these findings to broader populations. Additional exploration of the associations between PERHL scale scores and biomarkers of phthalate exposures should be pursued in larger samples with more diverse participant characteristics.

Overall, associating environmental health literacy scale scores with relevant biomarkers of exposure provides a means of validation of the environmental health literacy tool, and can contribute to future environmental health interventions by providing a quick, non-invasive tool to identify individuals who may be most at-risk for lower environmental health literacy and higher phthalate exposure.

## Data Availability

The data that support the findings of this study are available on request from the corresponding author, KST, and the PI of the ERGO Study, TJ-T. The data are not publicly available due to institutional restrictions that prohibit the sharing of data containing information that could compromise research participant privacy upon which participant consent was contingent.

## References

[1] Lucaccioni L, Trevisani V, Passini E, Righi B, Plessi C, Predieri B, et al. Perinatal exposure to phthalates: From endocrine to neurodevelopment effects. Int J Mol Sci. 2021;22:4063. 33920043 10.3390/ijms22084063 PMC8070995

[2] Corbasson I, Hankinson SE, Iii EJS, Reeves KW Urinary bisphenol-A, phthalate metabolites and body composition in US adults, NHANES 1999 – 2006. Int J Environ Health Res. 2016;26:606–17. 10.1080/09603123.2016.1233524 27643383

[3] Hauser R, Calafat AM. Phthalates and human health. Occup Environ Med. 2005;62:806–18. 10.1136/oem.2004.017590 PMC1740925 16234408

[4] Zhang Y, Mustieles V, Yland J, Braun JM, Williams PL, Attaman JA, et al. Association of parental preconception exposure to phthalates and phthalate substitutes with preterm birth. JAMA Netw Open. 2020;3:e202159. 32259265 10.1001/jamanetworkopen.2020.2159 PMC7139277

[5] Sharma S, Ashley JM, Hodgson A, Nisker J. Views of pregnant women and clinicians regarding discussion of exposure to phthalate plasticizers. Reprod Health. 2014;11:1–8. 24952638 10.1186/1742-4755-11-47 PMC4079618

[6] Phthalates. America's Children and the Environment. Third Edition, Updated August 2017. United States Environmental Protection Agency. 168–71. https://www.epa.gov/sites/default/files/2015-06/documents/ace3_2013.pdf.

[7] https://www.acog.org/clinical/clinical-guidance/committee-opinion/articles/2021/07/reducing-prenatal-exposure-to-toxic-environmental-agents Gynecologists TAC of O and. Committee Opinion. 2021 [cited 2021 Sep 30]. Reducing Prenatal Exposure to Toxic Environmental Agents | ACOG.

[8] Shaffer RM, Ferguson KK, Sheppard L, James-Todd T, Butts S, Chandrasekaran S, et al. Maternal urinary phthalate metabolites in relation to gestational diabetes and glucose intolerance during pregnancy. Environ Int. 2019;123:588–96. 30622083 10.1016/j.envint.2018.12.021 PMC6347428

[9] James-Todd T, Ponzano M, Bellavia A, Williams PL, Cantonwine DE, Calafat AM, et al. Urinary phthalate and DINCH metabolite concentrations and gradations of maternal glucose intolerance. Environ Int. 2022;161:107099. 10.1016/j.envint.2022.107099 PMC10723583 35085932

[10] Yang TC, Jovanovic N, Chong F, Worcester M, Sakhi AK, Thomsen C, et al. Interventions to reduce exposure to synthetic phenols and phthalates from dietary intake and personal care products: a scoping review. current environmental health reports. Springer Science and Business Media Deutschland GmbH; 2023 10.1007/s40572-023-00394-8 PMC10300154 36988899

[11] Risotto SP. Dietary intervention and DEHP reduction. Environ Health Perspect. 2011;119:a380. 21885374 10.1289/ehp.1103852 PMC3230409

[12] Harley KG, Kogut K, Madrigal DS, Cardenas M, Vera IA, Meza-Alfaro G, et al. Reducing phthalate, paraben, and phenol exposure from personal care products in adolescent girls: Findings from the hermosa intervention study. Environ Health Perspect. 2016;124:1600–7. 26947464 10.1289/ehp.1510514 PMC5047791

[13] Finn S, O’Fallon L. The emergence of environmental health literacy - from its roots to its future potential. Environ Health Perspect. 2017;125:495–501. https://ehp.niehs.nih.gov/wp-content/uploads/125/4/ehp.1409337.alt.pdf. 26126293 10.1289/ehp.1409337 PMC5382009

[14] Gray K. From content knowledge to community change: a review of representations of environmental health literacy. Int J Environ Res Public Health. 2018;15:466. 29518955 10.3390/ijerph15030466 PMC5877011

[15] Hoover AG. Defining Environmental Health Literacy. In: Finn S, O’Fallon L, editors. Environmental Health Literacy. 1st ed. Durham, North Carolina: Springer; 2019. p. 3–18.

[16] Tomsho K, Quinn M, Adamkiewicz G, James-Todd T. Development of a Phthalate Environmental Reproductive Health Literacy (PERHL) Scale. Environ Health Perspect. 2024;132:47013. 10.1289/EHP13128 PMC11050996 38669179

[17] Stanifer S, Hoover AG, Rademacher K, Rayens MK, Haneberg W, Hahn EJ. Citizen science approach to home radon testing, environmental health literacy and efficacy. Citiz Sci. 2022;7:26. 36845873 10.5334/cstp.472 PMC9949773

[18] Dellinger MJ, Pingatore N, Chelius T, Visotcky A, Sparapani R, Ripley M. Environmental health literacy for Anishinaabe (Great Lakes Native American) fish consumers: A randomized control trial. Environ Res. 2022;212:113335. 35447154 10.1016/j.envres.2022.113335

[19] Chan M, Preston EV, Fruh V, Quinn MR, Hacker MR, Wylie BJ, et al. Use of personal care products during pregnancy and birth outcomes – A pilot study. Environ Res. 2023;225:115583. 10.1016/j.envres.2023.115583 PMC10153796 36868449

[20] Thaweethai T, Soetan Z, James K, Florez JC, Powe CE. Distinct insulin physiology trajectories in euglycemic pregnancy and gestational Diabetes Mellitus. Diabetes Care. 2023;46:2137–46. 37126832 10.2337/dc22-2226 PMC10698215

[21] Samandar E, Silva MJ, Reidy JA, Needham LL, Calafat AM. Temporal stability of eight phthalate metabolites and their glucuronide conjugates in human urine. Environ Res. 2009;109:641–6. 19272594 10.1016/j.envres.2009.02.004

[22] Ferguson KK, McElrath TF, Ko YA, Mukherjee B, Meeker JD. Variability in urinary phthalate metabolite levels across pregnancy and sensitive windows of exposure for the risk of preterm birth. Environ Int. 2014;70:118–24. 24934852 10.1016/j.envint.2014.05.016 PMC4104181

[23] Fruh V, Preston EV, Quinn MR, Hacker MR, Wylie BJ, O’Brien K, et al. Urinary phthalate metabolite concentrations and personal care product use during pregnancy – Results of a pilot study. Sci Total Environ. 2022;835:155439. 35469886 10.1016/j.scitotenv.2022.155439 PMC11040873

[24] Boeniger MF, Lowry LK, Rosenberg J. Interpetation of urine results used to assess chemical exposure with emphasis on creatinine adjustments_a review. Am Ind Hyg Assoc J. 1993;54:615–27. 8237794 10.1080/15298669391355134

[25] Kim JH, Moon N, Heo SJ, Kwak JM, Effects of environmental health literacy-based interventions on indoor air quality and urinary concentrations of polycyclic aromatic hydrocarbons, volatile organic compounds, and cotinine: A randomized controlled trial. Atmos Pollut Res. 2024;15:101965.

[26] Finn S, O’Fallon L. The emergence of environmental health literacy—from its roots to its future potential. Environ Health Perspect. 2017;125:495–501. 26126293 10.1289/ehp.1409337 PMC5382009

[27] Boronow KE, Cohn B, Havas L, Plumb M, Brody JG. The effect of individual or study-wide report-back on knowledge, concern, and exposure-reducing behaviors related to endocrine-disrupting chemicals. Environ Health Perspect. 2023;131:97005. 37682721 10.1289/EHP12565 PMC10489892

